# Interaction between interleukin-1β and type-1 cannabinoid receptor is involved in anxiety-like behavior in experimental autoimmune encephalomyelitis

**DOI:** 10.1186/s12974-016-0682-8

**Published:** 2016-09-02

**Authors:** Antonietta Gentile, Diego Fresegna, Alessandra Musella, Helena Sepman, Silvia Bullitta, Francesca De Vito, Roberta Fantozzi, Alessandro Usiello, Mauro Maccarrone, Nicola B. Mercuri, Beat Lutz, Georgia Mandolesi, Diego Centonze

**Affiliations:** 1 Laboratory of Neuroimmunology and Synaptic Transmission, IRCCS Fondazione Santa Lucia, Centro Europeo di Ricerca sul Cervello (CERC), 00143 Rome, Italy; 2 Department of Systems Medicine, Tor Vergata University, 00133 Rome, Italy; 3 Unit of Neurology and of Neurorehabilitation, IRCCS Istituto Neurologico Mediterraneo Neuromed, 86077 Pozzilli, IS Italy; 4 Behavioural Neuroscience Laboratory, CEINGE Biotecnologie Avanzate, 80145 Naples, Italy; 5 Department of Environmental, Biological and Pharmaceutical Sciences and Technologies, Second University of Naples (SUN), Caserta, Italy; 6 Centro di Ricerca Integrata, Facoltà di Medicina e Chirurgia, Università Campus Bio-Medico, 00128 Rome, Italy; 7 Institute of Physiological Chemistry, University Medical Center of the Johannes Gutenberg University, 55128 Mainz, Germany

**Keywords:** Type-1 cannabinoid receptor, Striatum, Interleukin-1β, Anxiety, Experimental autoimmune encephalomyelitis

## Abstract

**Background:**

Mood disorders, including anxiety and depression, are frequently diagnosed in multiple sclerosis (MS) patients, even independently of the disabling symptoms associated with the disease. Anatomical, biochemical, and pharmacological evidence indicates that type-1 cannabinoid receptor (CB1R) is implicated in the control of emotional behavior and is modulated during inflammatory neurodegenerative diseases such as MS and experimental autoimmune encephalomyelitis (EAE).

**Methods:**

We investigated whether CB1R could exert a role in anxiety-like behavior in mice with EAE. We performed behavioral, pharmacological, and electrophysiological experiments to explore the link between central inflammation, mood, and CB1R function in EAE.

**Results:**

We observed that EAE-induced anxiety was associated with the downregulation of CB1R-mediated control of striatal GABA synaptic transmission and was exacerbated in mice lacking CB1R (CB1R-KO mice). Central blockade of interleukin-1β (IL-1β) reversed the anxiety-like phenotype of EAE mice, an effect associated with the concomitant rescue of dopamine (DA)-regulated spontaneous behavior, and DA-CB1R neurotransmission, leading to the rescue of striatal CB1R sensitivity.

**Conclusions:**

Overall, results of the present investigation indicate that synaptic dysfunction linked to CB1R is involved in EAE-related anxiety and motivation-based behavior and contribute to clarify the complex neurobiological mechanisms underlying mood disorders associated to MS.

## Background

Multiple sclerosis (MS) is the most common cause of neurological disability in young adults, affecting 1 in 800–1000 people in Western countries. Mood disturbances are frequent in MS, even in its early phases and in the absence of physical disability [1, 2].

Rather than merely representing a subjective reaction to a chronic and potentially disabling disease, anxiety and depression in MS are increasingly recognized to follow the effect of the inflammatory milieu on neuronal function and connectivity [3–6], thus sharing with the inflammatory neurodegenerative process of the disease of some neurobiological underpinnings. For example, proinflammatory cytokines such as interleukin-1β (IL-1β) and tumor necrosis factor (TNF), released during MS attacks, have been implicated both in delayed neurodegeneration in MS brains and in mood alterations [3], therefore suggesting common determinants for both phenomena. Studies in the experimental autoimmune encephalomyelitis (EAE), the most characterized murine model of MS, have clearly highlighted the independence of behavioral alterations from motor disability and, most notably, the involvement of cytokines [7–10].

Type-1 cannabinoid receptor (CB1R) are crucial regulators of both excitatory and inhibitory synaptic transmission in different brain areas [11, 12], including the striatum [13, 14] and the brain area involved in MS [15]. CB1R plays a substantial role in MS disease course [16–18] and in mood control [19]. Reduced CB1R signaling in mice lacking the CB1R gene (CNR1), in fact, results in more severe motor deficits and synaptic pathology linked to neurodegenerative damage after EAE [20, 21], and human subjects carrying a genetic variant of CNR1 associated with reduced CB1R protein expression have higher risk of progressive MS course [22], and more severe relapsing MS disease course [23], and neurodegenerative damage [24]. On the other hand, endocannabinoid signaling enhancement has antidepressant and anxiolytic actions in humans [25] and in rodents [26, 27], and genetic or pharmacological blockade of CB1R promotes depression- and anxiety-like behavior in humans [28, 29] and in rodents [30–32].

Whether CB1R plays a role in MS-associated mood alterations is however entirely speculative. To answer this question, we investigated the link between CB1R function and the emotional consequences of brain inflammation in EAE mice. In particular, we addressed the link between CB1R function and IL1-β effects at GABA synapses in the striatum of EAE mice. Indeed, IL1-β has been previously demonstrated to control the sensitivity of CB1R on GABAergic transmission and to induce anxiety-like behavior in naïve mice [33]. Moreover, we have recently shown that IL1-β is implicated in depressive-like behavior in EAE mice [10].

Our results point to CB1R as involved in EAE-associated anxiety and establish a previously unrecognized link between mood alterations and IL-1β-dependent inflammatory synaptic dysfunction in EAE and, possibly, MS.

## Methods

### Animals

The subjects in this study were 7–8-week-old female mice, C57BL/6N, obtained from Charles-River (Italy) and CNR-EMMA Mouse Clinic facility (Monterotondo-Rome, Italy). Animals were randomly assigned to standard cages, with four to five animals per cage, and kept at standard housing conditions with a light/dark cycle of 12 h and free access to food and water. Only minipump-implanted mice were housed in isolated cages endowed with special bedding (TEK-FRESCH, Harlan) in order to avoid skin infections around the surgical scar. Since 1 week before immunization, all animals were kindly handled once a day to reduce the stress induced by operator manipulation during behavioral experiments.

All experiments were carried out in accordance with the Guide for the Care and Use of Laboratory Animals and the European Communities Council Directive of 24 November, 1986 (86/609/EEC).

### EAE induction and clinical evaluation

Chronic EAE was induced in 7–8-week animals as previously described [10, 34]. Furthermore, EAE was induced in female mice totally lacking CB1Rs (CB1R-KO) [35] and respective wild-type (WT) littermate controls. Mice were injected subcutaneously at the flanks with 200 μg of myelin oligodendrocyte glycoprotein 35-55 (MOG_35-55_) emulsion to induce EAE by active immunization. The emulsion was prepared under sterile conditions using MOG_35-55_ (>85 % purity, Espikem, Florence, Italy) in 300 μl of complete Freund’s adjuvant (CFA, Difco, Lawrence, KS, USA) containing *Mycobacterium tuberculosis* (8 mg/ml; strain H37Ra, Difco) and emulsified with phosphate-buffered saline (PBS). All animals were injected with 500 ng pertussis toxin (Sigma, St. Louis, MO, USA) intravenously on the day of immunization and 2 days later. Control animals received the same treatment as EAE mice without the immunogen, MOG peptide, including complete CFA and Pertussis toxin (referred to as “CFA”). Animals were daily scored for clinical symptoms of EAE, according to the following scale: 0 = healthy; 1 = flaccid tail; 2 = ataxia and/or paresis of hindlimbs; 3 = paralysis of hindlimbs and/or paresis of forelimbs; 4 = tetraparalysis; and 5 = moribund or death due to EAE. Intermediate clinical signs were scored by adding 0.5 value [10, 35]. The presymptomatic phase was kept in the range 8–11 days post immunization (dpi), before the onset day, when immunized animals showed the first clinical manifestation (12 dpi), as previously shown [34].

### Minipump implantation and continuous intracranial infusion

One week before immunization, mice were implanted with a minipump in order to allow continuous intracerebroventricular (icv) infusion of either vehicle or interleukin 1 receptor antagonist (IL1-ra) (150 ng/day; R&D Systems) for 4 weeks. Alzet osmotic minipumps (model 1004; Durect Corporation, Cupertino, CA) connected via catheter tube to intracranial cannula (Alzet Brain Infusion Kits 3) delivered vehicle or IL1-ra into the right lateral ventricle at a continuous rate of 0.11 μl/h. The coordinates used for icv minipump implantation were antero-posterior = −0.4 mm from the bregma; lateral = −1 mm; and depth: 2.5 mm from the skull [10, 36].

### In vivo amphetamine treatment

EAE mice were given intraperitoneal injection of amphetamine sulfate (5 mg/kg) in a volume of 10 ml/kg or vehicle [37] and after 24 h were sacrificed for both electrophysiological and western blot experiments. Control CFA and EAE mice received intraperitoneal injection of saline solution (NaCl 0.9 %). Each experimental group consisted of four to five animals (three sets of experiments).

### Behavioral assessment

Behavioral experiments were performed during the presymptomatic phase of the disease (8–9 dpi). The animals were tested during light period (9:00–12:00 am) in a dedicated room with a constant temperature (26 ± 1 °C). All tests were performed in different days with distinct groups of animals. Each session was preceded by at least 1 h habituation in the behavioral room.

#### LDT

The light/dark test (LDT) is based on the innate aversion of rodents to brightly lit areas [38]. The test apparatus consisted of an open white compartment (30 × 20 × 20 cm, 300 lux) joined by a 3 × 3-cm opening to a dark compartment (15 × 20 × 20 cm, 0 lux) which was painted black and covered with a lid. The anxiogenic nature of the white compartment was increased by additional illumination from a 60-W angle poise lamp placed 45 cm above the center of the apparatus. Mice were allowed to move freely between the two chambers with door open for 10 min. The score for the transition was assigned from the analysis of the video recordings, when the animal came out of the dark chamber with all four paws. The apparatus was cleaned with 10 % ethanol after each trial to effectively remove the scent of the previously tested animal. The time spent in each chamber (referring to the last 5 min of the test) was recorded by ViewPoint video tracking software.

#### NB

Nest building is a natural and instinctual behavior that involves species-typical sensorimotor actions important to the survival of the animal. These behaviors are dependent upon motivation [39]. To evaluate the quality of nest construction, mice were individually housed 1 h before the onset of dark phase in a clean cage overnight with no enrichment aside a pre-weighted roll of cotton in the cage-top food hopper. The morning after (9 am), the quality of the nest were evaluated using the following scoring system: (1) no nest, (2) platform-type nest consisting of a pallet on the floor of the cage, (3) bowl- or cupshaped nest with sides, or (4) bowl- or cup-shaped nest with sides and a cover [40]. An investigator blind to treatment and experimental group scored the quality of the nests.

### Electrophysiology

Mice were killed by cervical dislocation, and corticostriatal coronal slices (200 μm) were prepared from fresh tissue blocks of the brain with the use of a vibratome [10]. A single slice was transferred to a recording chamber and submerged in a continuously flowing artificial cerebrospinal fluid (ACSF) (34 °C, 2–3 ml/min) gassed with 95 % O2–5 % CO_2_. The composition of the control ACSF was (in mM) 126 NaCl, 2.5 KCl, 1.2 MgCl2, 1.2 NaH2PO4, 2.4 CaCl2, 11 Glucose, and 25 NaHCO3. The striatum could be readily identified under low power magnification, whereas individual neurons were visualized in situ using a differential interference contrast (Nomarski) optical system. This employed an Olympus BX50WI (Japan) non-inverted microscope with 40× water immersion objective combined with an infra-red filter, a monochrome CCD camera (COHU 4912), and a PC compatible system for analysis of images and contrast enhancement (WinVision 2000, Delta Sistem, Italy). Recording pipettes were advanced towards individual striatal cells in the slice under positive pressure and visual control (WinVision 2000, Delta Sistemi, Italy) and, on contact, tight GΩ seals were made by applying negative pressure. The membrane patch was then ruptured by suction and membrane current and potential monitored using an Axopatch 1D patch clamp amplifier (Molecular Devices, Foster City, CA, USA). Whole-cell access resistances measured in voltage clamp were in the range of 5–20 MΩ. Whole-cell patch clamp recordings were made with borosilicate glass pipettes (1.8 mm o.d.; 2–3 MΩ), in voltage-clamp mode, at the holding potential of −80 mV.

To study GABA-mediated spontaneous inhibitory postsynaptic currents (sIPSCs), the recording pipettes were filled with internal solution of the following composition (mM): 110 CsCl, 30 K^+^-gluconate, 1.1 EGTA, 10 HEPES, 0.1 CaCl_2_, 4 Mg-ATP, 0.3 Na-GTP. MK-801, and CNQX were added to the external solution to block, respectively, NMDA and non-NMDA glutamate receptors. Drugs were first dissolved in water or in DMSO (HU-210) and then in the ACSF to the desired final concentration. The concentrations of the various drugs were chosen according to previous in vitro studies on corticostriatal brain slices [33, 41] and were as follows (in μM): 10 CNQX, 25 MK-801, and 1 HU-210 (Tocris Bioscience).

Synaptic events were stored by using P-CLAMP 9 (Axon Instruments) and analyzed offline on a personal computer with Mini Analysis 5.1 (Synaptosoft, Leonia, NJ, USA) software. The detection threshold of sIPSCs was set at twice the baseline noise. The fact that no false events would be identified was confirmed by visual inspection for each experiment. Offline analysis was performed on spontaneous synaptic events recorded during fixed time epochs (1–2 min), sampled every 2–3 min (5–12 samplings) [34].

Only data from putative GABAergic medium spiny projection neurons (MSNs) were included in the present study and identified immediately after rupture of the GΩ seal, by evaluating their firing response to the injecting of depolarizing current (typically tonic, with little or no adaptation).

One to five cells per animal were recorded. For each type of experiment and time-point, at least four mice per group were employed. Electrophysiological results from neurons recorded from the same animal were treated as a separate sample and averaged before calculating statistics. One animal per day was used for the electrophysiological experiment. Throughout the text “n” refers to the number of cells, unless otherwise specified.

### Striatal total protein extracts preparation and WB

At least 4 animals per group were included in all western blot (WB) experiments. Mice were sacrificed through cervical dislocation, and both left and right striata were quickly removed and frozen until use. Tissues were homogenized in RIPA buffer plus protease and phosphatase inhibitors cocktail (SIGMA) as previously described [10, 36].

Primary antibodies were used as following: mouse anti-β-actin (1:20,000, 1 h RT; Sigma-Aldrich), rabbit anti-dopamine (DA)- and cAMP-regulated phosphoprotein 32 kDa (DARPP32; 1:50,000, 1 h RT; Abcam), rabbit anti-p-Th34-DARPP32 (1:1000, overnight 4 °C; Merck), goat anti-p-Ser316-CB1R (1:200, overnight 4 °C; SantaCruz), and goat anti-CB1R (1:200, overnight 4 °C; SantaCruz). Membranes were incubated with the following secondary antibodies: anti-mouse IgG HRP (1:10,000; GE Healthcare, formerly Amersham Biosciences), anti-rabbit IgG HRP (1:2000; GE Healthcare, formerly Amersham Biosciences), anti-goat IgG HRP (1:2000; GE Healthcare, formerly Amersham Biosciences), and diluted in 1 % milk for 1 h at RT. Blots were stripped with Restore Western Blot Stripping Buffer (Thermo Scientific), after detection of phospho-sites. Complete stripping was assessed by incubation with proper secondary antibody immunodetection was performed by ECL reagent (Amersham) and membrane was exposed to film (Amersham). Densitometric analysis of protein levels was performed by NIH ImageJ software (http://rsb.info.nih.gov/ij/). Phospho-CB1R band densitometry was normalized respect to β-actin, since in a different blot of the same samples, we did not detect changes in CB1R unphosphorylated protein, while DARPP32 phospho-protein levels were normalized to DARPP32 unphosphorylated protein to account for changes in the amount of DARPP32 unphosphorylated protein. WB results are presented as data normalized to control CFA values.

### RNA extraction and qPCR

Total RNA was extracted according to the standard miRNeasy Micro kit protocol (QIAGEN). The RNA quantity and purity were analyzed with NanoDrop 2000c spectrophotometer (Thermo Scientific). The quality of RNA was assessed by visual inspection of the agarose gel electrophoresis images. Next, 250 ng of total RNA was reverse-transcribed using high-capacity cDNA reverse transcription kit (Applied Biosystem) according to the manufacturer’s instructions and 20 ng of cDNA was amplified with SensiMix SYBR Hi-Rox Kit (Bioline; Meridian Life Science) in triplicate using the Applied Biosystem 7900HT Fast Real Time PCR system. Relative quantification was performed using the ΔΔCT method. β-actin was used as internal controls. The following primer sequences were used.

Brain-derived neurotrophic factor (BDNF) (NM_007540): 5′-ACCATAAGGACGCGGACTTGT-3′ (sense); 5′-AAGAGTAGAGGAGGCTCCAAAGG-3′ (antisense); β-actin (NM_007393): 5′-CCTAGCACCATGAAGATCAAGATCA-3′ (sense); and 5′-AAGCCATGCCAATGTTGTCTCT-3′ (antisense).

### Statistical analysis

Data were presented as mean ± SEM. Throughout the text, “n” refers to the number of animals, with the exception of electrophysiological experiments, where “n” refers to the number of the cells. Two-sample comparisons were carried out using the Student’s *T* test for parametric measures or Mann-Whitney for non-parametric variables, while multiple comparisons were made using one-way ANOVA followed by Tukey’s HSD or non-parametric Kruskal–Wallis test followed by Dunn’s comparisons. The main effects of the two conditions (genotype and EAE) on the dependent behavioral variables and the interactions genotype × EAE were analyzed by performing two-way ANOVAs. The significance level was established at *p* < 0.05.

## Results

### EAE-induced anxiety is associated with CB1R desensitization in the striatum

We investigated the anxiety-like phenotype associated with MOG-induced central inflammation in presymptomatic EAE mice. In these mice, we assessed anxious-depressive-like responses by using the LDT and the nest building (NB) paradigm, a motivation-based task [39].

**Fig. 1:**
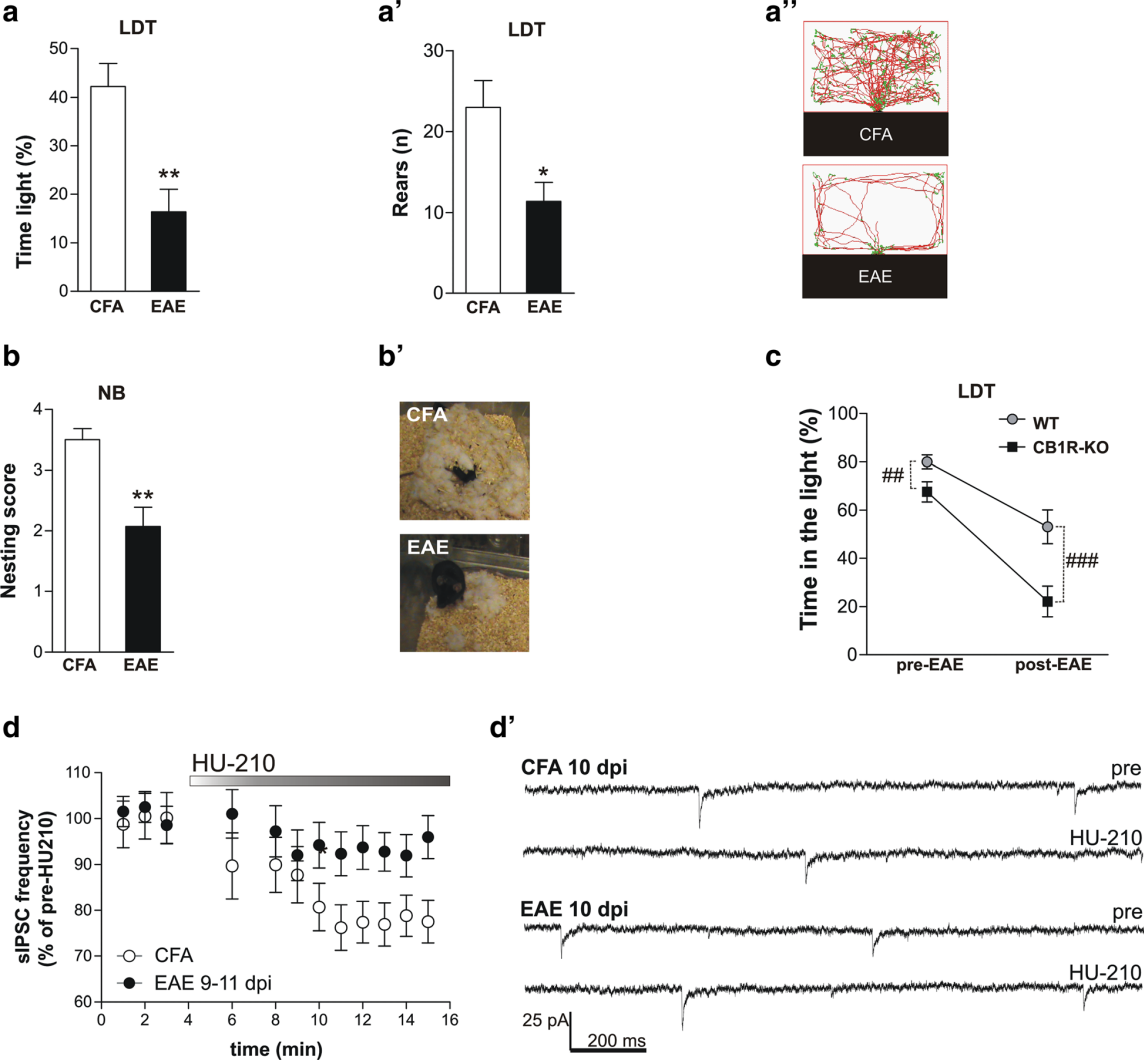
Anxiety-like behavior in EAE is associated to CB1R dysfunction in the striatum. **a** Exploratory behavior of EAE mice was investigated firstly by LDT confirmed increased anxiety-like behavior in EAE group as showed by time spent in the light zone of the task. **a**’ Rear episodes in EAE mice were severely reduced in comparison to CFA mice. **a**” Representative video-recording tracking of CFA and EAE mice performance in the light zone of the LDT apparatus. **b**, **b**’ EAE and CFA control mice were evaluated for their ability to construct nests (rated on a 4-point scale) to investigate motivation-based behavior. *Bar graph* shows a reduction in the quality of nest in EAE mice (**b**). **b**’. Representative photographs of nests built by CFA and EAE mice. **c**. The performance of CB1R-KO mice at the LD test (% of time spent in the bright compartment) revealed an anxiety-related behavior, which is heavily affected by EAE induction. **d**, **d**’ Bath application of the CB1R agonist HU210 on striatal slices induced sIPSC frequency reduction in CFA mice (*p* < 0.01). In EAE striatal slices, the effect of HU210 was abolished (*p* > 0.05) (**d**). Representative electrophysiological traces are depicted in **d**’. Values are means ± SEM. Statistical differences were analyzed by unpaired T-test or Mann-Whitney test (behavior) and by paired T-test (electrophysiology). *p < 0.05, **p < 0.01. Two-way ANOVA analysis for genotype factor: ##*p* < 0.01, ### *p* < 0.0001

Significant differences between EAE and controls emerged at the LDT (Fig. 1a–a”), since the time spent in the light zone (EAE 16.42 ± 4.6 %; CFA: 42.21 ± 4.72 %; unpaired *T* test; *p* < 0.01; Fig. 1a) and the number of rearing episodes during LDT (CFA: 23 ± 3.32, *n* = 8; EAE: 11.38 ± 2.33, *n* = 8, unpaired *T* test: *p* < 0.05; Fig. 1a’) were reduced in EAE, indicating both anxiety-like behavior and reduced motivation-based activity, respectively, in accordance with our previous findings [8]. Next, we measured goal-directed behavior of EAE and control mice by using the NB test. Nesting ability is a natural, instinctive motivation-driven behavior in rodents [39]. Murine NB skills have been found altered during inflammation challenge [42, 43] and, of note, in EAE mice during the acute phase of the disease independently of motor defects [10]. Preclinical EAE mice performed significantly worse than controls in terms of nesting score (CFA 3.5 ± 0.18; EAE 2.07 ± 0.31; Mann-Whitney non-parametric test *p* < 0.01) (Fig. 1b, b’), again indicating that EAE is associated with reduction of social behavior, which is a characteristic trait of sickness behavior, affecting EAE mice [44].

The endocannabinoid system (ECS) is deeply involved in mood control [25, 26]. We asked whether CB1Rs could be involved in EAE-behavioral syndrome. One week before immunization, we evaluated the spontaneous responses of WT and CB1R-KO mice at LDT, in order to monitor anxiety-like behavior under basal condition. After 2 weeks, during EAE (7–9 dpi), the mice were tested again for anxiety by means of LDT.

CB1R-KO mice spent less time in the lit compartment than WT mice (*n* = 8 for each groups; *F* = 13.54, *p* < 0.01), showing increased anxiety-related behavior as previously reported [30]. Such behavior was exacerbated by EAE induction. EAE induction had a substantial effect on behavioral performance, accounting for 47.01 % of total variance (*F* = 40.76, *p* < 0.0001), with a significant interaction with genotype (*F* = 4.403, *p* < 0.05) (Fig. 1c), suggesting the role of CB1Rs in anxiety-like behavior associated to EAE.

We have previously reported that the downregulation of CB1R-mediated control of GABA synapses in the striatum represents a reliable synaptic correlate of anxiety induced by chronic psychoemotional stress [45] or by a single intracerebroventricular injection of IL-1β [33]. Of note, in a previous study, we demonstrated that CB1R sensitivity on GABA synapses is lost in the striatum of EAE mice during the symptomatic phase of the disease [46]. We therefore assessed whether EAE-induced anxiety was also associated with alterations of CB1R function. Thus, control and EAE mice were sacrificed to study synaptic transmission in the striatum (9–11 dpi). Here, we found that application of HU210 (1 μM, 10 min) significantly reduced the frequency of sIPSCs in CFA mice (79.8 ± 3.5 %; *n* = 10; *p* < 0.01), while this CB1R agonist failed to alter GABA transmission in the presymptomatic EAE mice (96.3 ± 3.4 %; *n* = 9; *p* > 0.05), suggesting that this synaptic alteration is precocious and, more interestingly, is linked to behavioral alterations in EAE (Fig. 1d, d’).

Overall, these data indicate that CB1Rs are involved in EAE-mediated anxiety-like behavior occurring in the presymptomatic phase of the disease.

### Blockade of IL-1β signaling reduces anxiety, rearing defects, and CB1R dysfunction in EAE

To investigate the role of IL-1β in EAE-induced anxiety, we chronically inhibited IL-1β signaling in these mice by icv delivery of IL-1ra. We have previously shown that this preventive treatment reduced motor disability and inflammatory reaction in the cerebellum, the hippocampus, and the striatum of EAE mice during the symptomatic phase of the disease [10, 36, 47]. Moreover, increased IL1-beta expression in the striatum of EAE mice with preserved motor functions was associated to depressive-like and motivation-based behaviors observed in these mice [10]. Here, we investigated the anxious-like behavior of EAE mice treated with IL-1ra during the preclinical phase of EAE, when motor defects are not detectable.

**Fig. 2:**
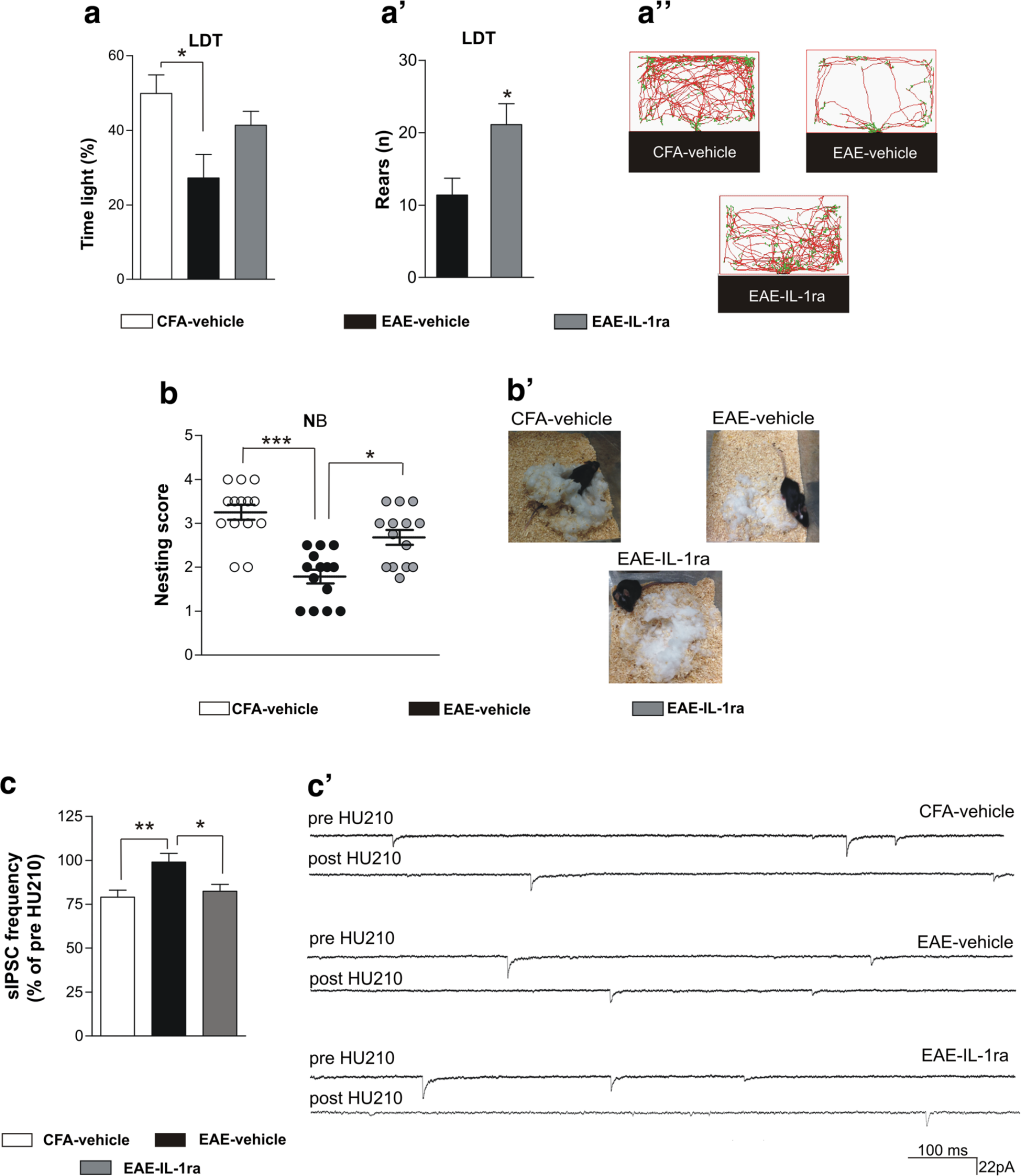
IL-1ra preventive treatment improves EAE behavioral syndrome and restores striatal CB1R function. **a**–**a**” At the LDT, the IL-1ra treatment improved the behavioral alterations of EAE mice; the time spent in the light zone, EAE-IL-1ra mice showed values similar to CFA-vehicle, although being not significantly different to both CFA and EAE-vehicle (**a**). **a**’ Vertical activity increased in EAE-IL-ra mice, indicating an ameliorated explorative response induced by IL1-ra treatment. Examples of LDT video-tracking are depicted in **a**”. **b**, **b**’ Consistently with the results obtained in non-minipump-implanted animals (shown in Fig. 1**b**, **b**’), the nesting score of EAE-vehicle mice was worse than CFA-vehicle animals and IL-1ra icv treatment corrected these behavioral alterations (**b**). Representative photographs in **b**’. **c**, **c**’. The lack of the depressant effect mediated by HU210 in EAE slices was rescued in EAE mice receiving in vivo treatment of IL-1ra by minipump implantation (**c**). In **c**’, there are examples of electrophysiological traces showing the lack of the effect of HU210 only in EAE-vehicle mice. Values are means ± SEM. Statistical differences were analyzed by one-way ANOVA for multiple comparisons (followed by Tukey HSD or Dunn's comparisons) or by unpaired T-test for rear numbers in LDT. *p < 0.05, **p < 0.01, ***p < 0.001

In the LDT, IL-1ra partially corrected the behavioral alterations of pre-symptomatic EAE mice. The time spent in the lit zone by EAE-IL-1ra mice was not different with respect to both CFA-vehicle and EAE-vehicle (CFA-vehicle 49.90 ± 4.98, EAE-vehicle 27.18 ± 6.34, EAE-IL-1ra 41.34 ± 3.76; one-way ANOVA post hoc comparisons: CFA-vehicle vs EAE-vehicle *p* < 0.05; CFA-vehicle vs EAE-IL-1ra *p* > 0.05; EAE-vehicle vs EAE-IL-1ra *p* > 0.05), although very similar to CFA-vehicle (Fig. 2a, a”). However, rearing activity during the LDT was fully corrected by icv IL-1ra delivery in EAE mice (EAE-IL-1ra: 21.13 ± 2.88, *n* = 8, vs EAE-VH: 11.38 ± 2.33, *n* = 8; *p* < 0.05; Fig. 2a’).

We next addressed the effects of the IL-1ra icv delivery in EAE mice at the NB test. Consistently with the results in non-minipump-implanted animals, the nesting score of EAE mice receiving icv vehicle (9 dpi, *n* = 14; 1.78 ± 0.15) was worse than CFA-vehicle animals (*n* = 14; 3.25 ± 0.17), and IL-1ra icv treatment corrected these behavioral alterations (2.67 ± 0.16; *n* = 14; non-parametric Kruskal–Wallis test followed by Dunn’s comparisons: CFA-vehicle vs EAE-vehicle *p* < 0.001; CFA-vehicle vs EAE-IL-1ra *p* > 0.05; EAE-vehicle vs EAE-IL-1ra *p* < 0.05) (Fig. 2b, b’).

We performed electrophysiological measurements of striatal CB1R sensitivity in these mice 1 week after the behavioral tests in order to recover the effect of the stress induced by behavioral manipulation on endocannabinoid system [48]. Of note, the loss of CB1R sensitivity on GABA synapses is a synaptic dysfunction that occurs throughout the disease progression ([46]; present paper]) and IL-1ra treatment was previously shown to ameliorate both the depressive-like behavior of EAE mice in the acute phase of the disease and the dopaminergic dysfunction affecting the striatum [10].

We observed that the preventive and central treatment with IL-1ra rescued the physiological response of CB1R to HU210 (% of sIPSC frequency pre-HU210: CFA-vehicle 81.60 ± 5.6, *n* = 8; EAE-vehicle 108.4 ± 5.8, *n* = 9; EAE-IL-1ra 82.4 ± 3.9, *n* = 10; one-way ANOVA post hoc comparisons: CFA-vehicle vs EAE-vehicle *p* < 0.01; EAE-vehicle vs EAE-IL-1ra *p* < 0.05) (Fig. 2c, c’).

Altogether, these data are consistent with the idea that EAE anxiety-like behavior is linked to IL-1β-mediated CB1R dysfunction.

### Dopamine system is involved in EAE-induced alteration of striatal CB1R function

Striatal CB1R function is regulated by the DA system. Accordingly, pharmacological enhancement of DA signaling in vivo sensitizes CB1R [13] and DA receptor inhibition blocks them [49]. Recent biochemical and electrophysiological experiments demonstrated impaired DA transmission in the striatum of EAE mice [10], raising the possibility that also CB1R dysfunction is mediated by defective DA signaling in this neuroinflammatory condition. Of note, genetic and pharmacological blockade of DA pathway is known to reduce rearing activity in mice [50–52]. The reduced rearing activity observed in EAE mice confirmed defective DA signaling in EAE mice [10] and suggested that CB1R dysfunction could indeed be secondary to this alteration in EAE.

**Fig. 3:**
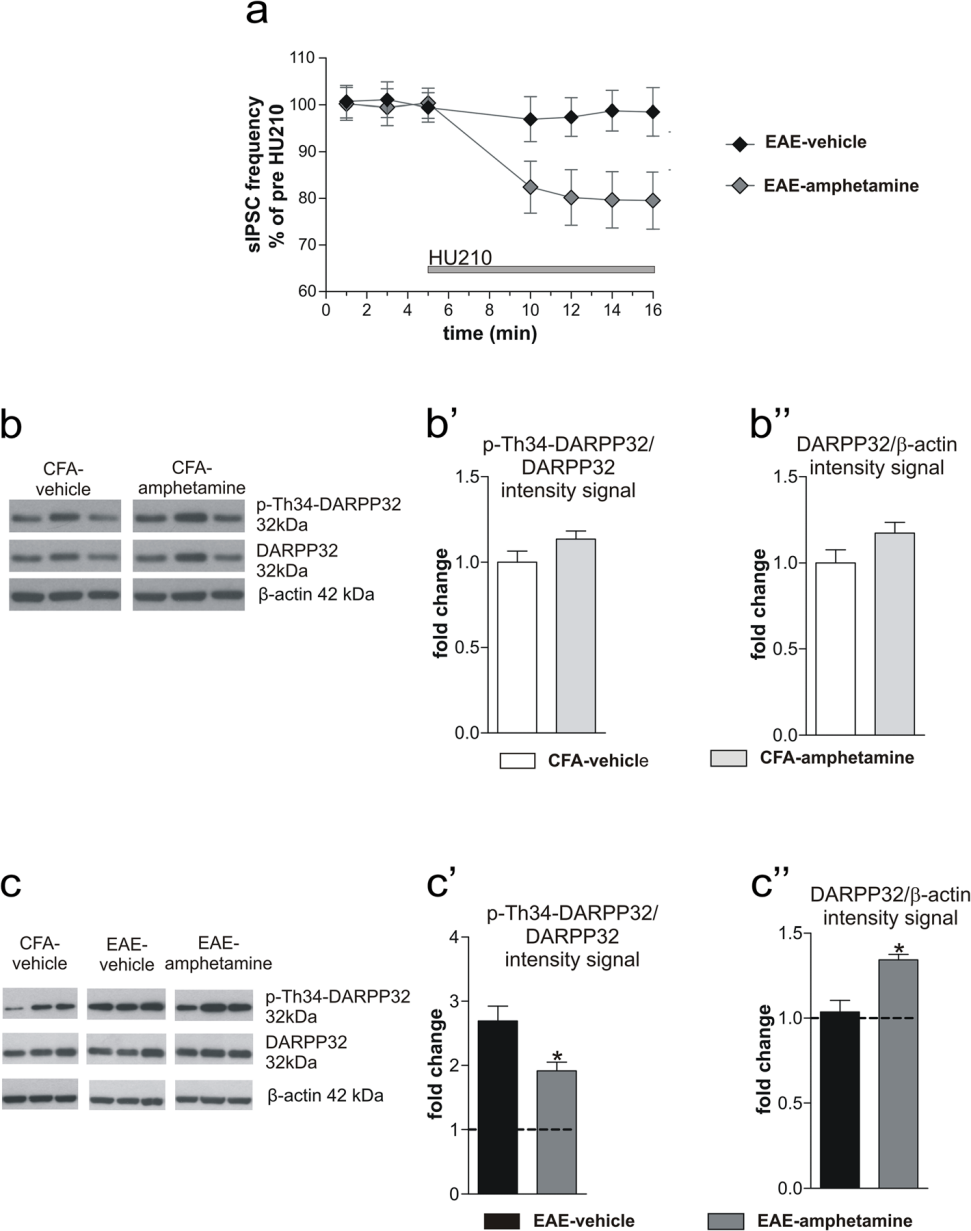
CB1R function is linked to dopamine system in the EAE striatum. **a** The inhibitory effect of HU210 on sIPSC frequency of striatal neurons was completely restored in EAE mice receiving i.p. injection of amphetamine (*p* < 0.05). **b**–**b**” Amphetamine treatment did not affect DARPP32 phosphorylation in the striatum of control CFA mice, as shown by the WB image in **b** and the densitometric analysis in **b’**. The treatment did not change the amount of unphosphorylated protein, as shown in **b** and **b**”. **c**–**c**” Representative WB of striatal protein extracts from EAE-vehicle and EAE-amphetamine mice: the densitometric analysis of the bands displays an upregulation of Th34-DARPP32 in EAE-vehicle samples and a partial recovery of such phosphorylation in EAE-amphetamine group, as shown by graph in **c**’. Amphetamine treatment induced a significant upregulation of DARPP32 expression in EAE striatum (**c**”). WB data are normalized to CFA values. Values are means ± SEM. Statistical differences were analyzed by paired Student’s *T* test (electrophysiology) and one-way ANOVA followed by Tukey HSD (WB). *p < 0.05 EAE-amphetamine vs EAE-vehicle

We tested therefore the hypothesis that defective DA transmission could be responsible of CB1R downregulation in EAE by evaluating striatal CB1R function in EAE mice treated in vivo with amphetamine, to favor DA release from DA nerve terminals. In striatal brain slices prepared from these animals, HU210 caused the expected inhibition of sIPSC frequency (EAE-amphetamine: 80.06 ± 3.2 %, *n* = 11, paired *T* test *p* < 0.05; EAE-vehicle: 97.8 ± 3.6, *n* = 10; paired *T* test *p* > 0.05), confirming the rescue of CB1R function by DA system stimulation in EAE mice (Fig. 3a).

We have recently found that phosphorylation at threonine 34 (P-Th34) of the DA- and cAMP-regulated phosphoprotein 32 kDa (DARPP32), marker of striatal MSNs, is increased in EAE, possibly due to unbalanced signaling through D1- and D2-like receptors [10]. By WB experiments, we checked the phosphorylation of DARPP32 at Th34 site after in vivo treatment with amphetamine to assess whether amphetamine could interfere with DA signaling in MSNs. Amphetamine treatment in CFA mice did not change neither the extent of DARPP32 phosphorylation at Th34 (p-Th34-DARPP32/DARPP32: CFA-vehicle 1 ± 0.06, *n* = 6; CFA-amphetamine 1.13 ± 0.04, *n* = 7; unpaired *T* test *p* > 0.05; Fig. 3b, b’), nor the total amount of DARPP32 (DARPP32/β-actin ratio: CFA-vehicle 1 ± 0.07 *n* = 6; CFA-amphetamine 1.17 ± 0.06, *n* = 7; unpaired *T* test *p* > 0.05; Fig. 3b–b”). Amphetamine reduced, although not completely, the hyper-phosphorylation of Th34-DARPP32 induced by EAE (p-Th34-DARPP32/DARPP32 ratio: CFA-vehicle 1 ± 0.25, EAE-vehicle 2.7 ± 0.23, EAE-amphetamine 1.91 ± 0.13; CFA-vehicle *n* = 5 vs EAE-vehicle *n* = 5, one-way ANOVA post hoc comparison: *p* < 0.001; CFA-vehicle vs EAE-amphetamine *n* = 4; one-way ANOVA post hoc comparison: *p* < 0.05; Fig. 3c, c’). However, the amphetamine treatment induced a slight increase in the expression of total DARPP32 in the EAE striatum (DARPP32/β-actin ratio: CFA-vehicle 1 ± 0.07, EAE-vehicle 1.03 ± 0.07, EAE-amphetamine 1.34 ± 0.03; one-way ANOVA: CFA-vehicle vs EAE-amphetamine *p* < 0.01; EAE-vehicle vs EAE-amphetamine *p* < 0.05) (Fig. 3c–c”), which accounted for the quantification of p-Th34-DARPP32, suggesting the triggering of compensatory mechanism induced by amphetamine to cope with the EAE-induced overstimulation of DA system.

**Fig. 4:**
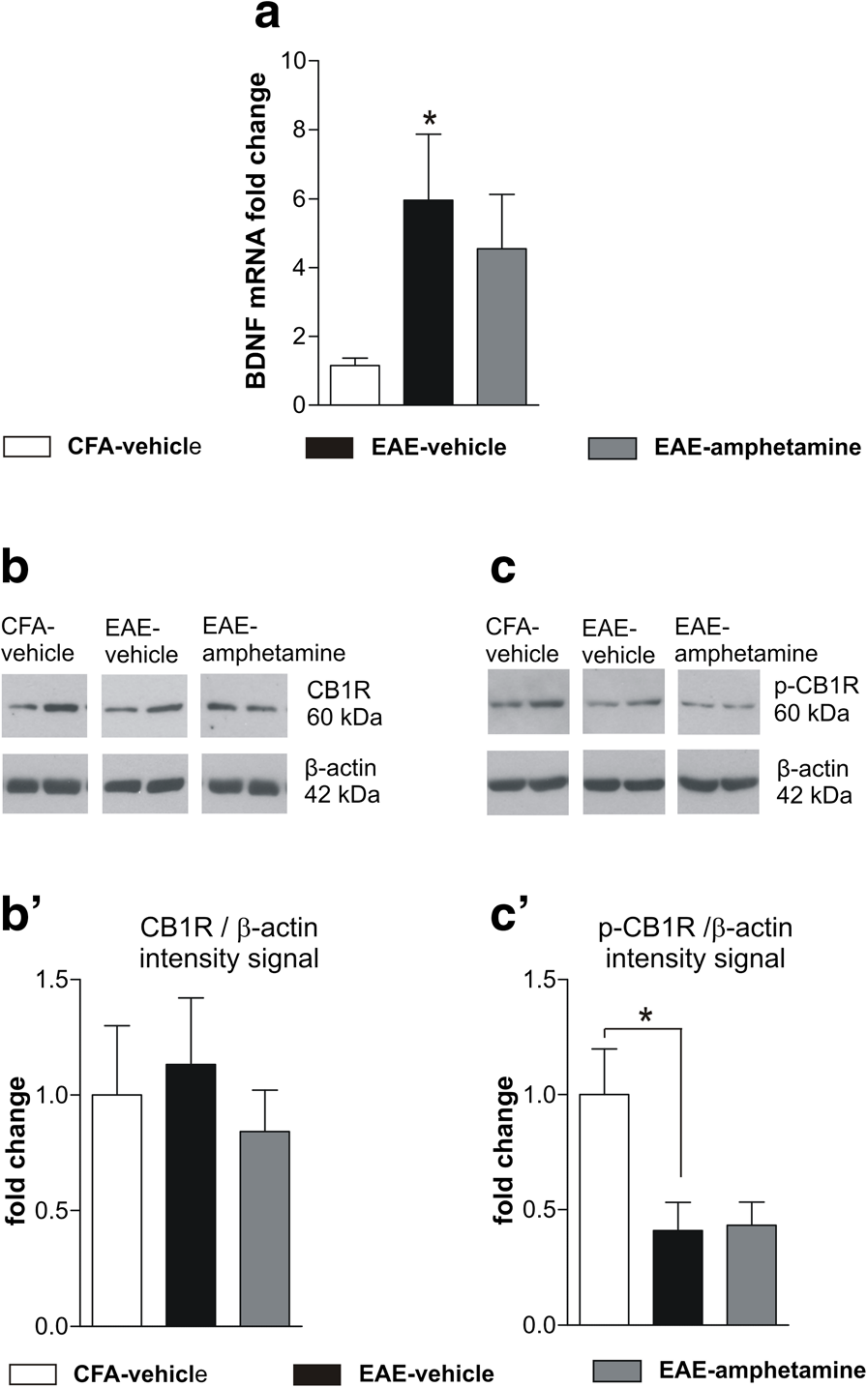
BDNF and CB1R signaling in EAE striatum. **a** BDNF mRNA in EAE-vehicle striatum is upregulated with respect to control CFA-vehicle (*p* < 0.05). Such alteration is not corrected by amphetamine treatment. **b** shows WB of striatal lysates of CFA-vehicle, EAE-vehicle, and EAE-amphetamine. CB1R levels in the striatum are not affected by EAE, as indicated by WB analysis of protein lysates (**b**’). Amphetamine given 24 h before the sacrifice of the animals did not affect the total amount of CB1R in the EAE striatal lysates. CB1R signal was normalized to β-actin bands. WB data are normalized to CFA values. **c**, Representative WB of lysates from CFA-vehicle, EAE-vehicle, and amphetamine-vehicle probed with antibody specific for the carboxy-terminal phosphorylation of CB1R (p-Ser316): the histogram in **c**’ indicates a remarkable reduction of CB1R phosphorylation, normalized to β-actin, which is not modulated by amphetamine treatment. Values are means ± SEM. Statistical differences were analyzed by one-way ANOVA followed by Tukey HSD (WB and qPCR). *p < 0.05 EAE-vehicle vs CFA-vehicle

We investigated the possible mechanisms underpinning the link between the CB1R- and DA-mediated neurotransmission. We first assessed the expression of the BDNF, which was previously shown to be a downstream effector of D2Rs in the modulation of CB1R in the mouse striatum [49]. By qPCR, we found that BDNF transcript was significantly increased in the striatum of EAE-vehicle mice (*n* = 6, fold change mRNA: 5.95 ± 1.90) compared to CFA-vehicle mice (*n* = 8, fold change mRNA: 1.15 ± 0.22), and that amphetamine treatment was not able to re-establish normal levels of BDNF expression (*n* = 6, fold change mRNA: 5.23 ± 1.69; one-way ANOVA Tukey post hoc comparisons: CFA-vehicle vs EAE-vehicle *p* < 0.05, CFA-vehicle vs EAE-amphetamine *p* > 0.05, EAE-vehicle vs EAE-amphetamine *p* > 0.05; Fig. 4a). This result indicated that BDNF modulation was not involved in the observed recovery of neurotransmission by amphetamine.

We next asked whether amphetamine treatment could modify the phosphorylation status of CB1R. CB1R undergoes phosphorylation at serine 316 (p-Ser316), and such phospohorylation has been linked to the internalization of the receptor-ligand complex [53]. WB experiments showed that CB1R levels did not differ among the three experimental groups EAE-vehicle (*n* = 5, CB1R/β-actin ratio: 1 ± 0.02), CFA-vehicle (*n* = 5, CB1R/β-actin ratio: 1.13 ± 0.28), and EAE-amphetamine (*n* = 4; CB1R/β-actin ratio: 0.84 ± 0.18; one-way ANOVA Tukey post hoc comparison: *p* > 0.05 for all comparisons; Fig. 4b, b’). Conversely, compared to control (CFA-vehicle *n* = 5, p-Ser316-CB1R/β-actin ratio: 1 ± 0.19) EAE striatal lysates (*n* = 5; p-Ser316-CB1R/β-actin ratio: 0.40 ± 0.12) showed a strong downregulation of the p-Ser316-CB1R, which was not recovered by amphetamine treatment (*n* = 4, p-Ser316-CB1R/β-actin ratio: 0.43 ± 0.10; one-way ANOVA Tukey post hoc comparisons: CFA-vehicle vs EAE-vehicle *p* < 0.05, CFA-vehicle vs EAE-amphetamine *p* > 0.05, EAE-vehicle vs EAE-amphetamine *p* > 0.05, Fig. 4c, c’), excluding this mechanism for amphetamine-mediated recovery of CB1R sensitivity on GABA terminals.

## Discussion

The results of the present investigation suggest the involvement of CB1Rs in the anxious phenotype of EAE mice. EAE-induced anxiety was in fact associated with a dramatic downregulation of CB1R-mediated presynaptic inhibition of GABA transmission in the striatum, a brain area increasingly recognized to play a substantial role in anxiety control in humans and rodents [54, 55] and affected in both EAE [34] and MS [15]. Anxiety was exacerbated in EAE mice lacking CB1Rs, and both the anxiety-like phenotype and striatal CB1R sensitivity were rescued in EAE mice by central inhibition of IL-1β signaling with IL-1ra. Moreover, CB1R function was restored by amphetamine treatment, providing further evidence for the cannabinoid-dopamine interaction in the striatum of EAE mice.

Despite the high prevalence and severity of mood disturbances in the MS population, depression and anxiety are generally underestimated and undertreated [56] and poor knowledge of the pathophysiological mechanisms of mood alteration in MS explains, at least in part, the scarce attention paid to this serious comorbid condition. Several studies in the EAE model have highlighted the independence of behavioral alterations in EAE mice from motor disability and linked these to the effects of pro-inflammatory cytokines, like TNF and IL1-β, in different brain circuits, like the amygdala, the hippocampus, and the striatum [7–10]. TNF and IL-1β, like other pro-inflammatory cytokines, have been convincingly associated with mood disorders both in humans [57, 58] and rodents [59], and their levels increased in serum and CSF of MS patients [3, 6, 60], and in EAE brains [8–10, 36].

We previously associated EAE anxious-like behavior to TNF-induced altered glutamatergic transmission in the striatum [8]. The present investigation links the anxiety-like behavior of presymptomatic EAE mice to IL-1β-induced CB1R dysfunction on GABA synapses. Overall, the studies coming from our and other labs indicate that the behavioral syndrome associated to EAE reflects the complex and parallel action of proinflammatory molecules on specific synaptic targets.

In this respect, here, we showed that the loss of CB1R sensitivity on GABA synapses, previously described in the symptomatic phase of the disease [46], occurs early, before the appearance of motor symptoms, raising the possibility that this synaptic defect could be involved in EAE-anxiety-like behavior. In mice lacking CB1R, EAE caused a significant increase of anxiety after EAE in both WT and CB1R-KO mice at the LDT. CB1R-KO mice, however, developed a much more marked anxiety-like behavior in the preclinical phase of EAE compared to their WT counterpart, indicating high vulnerability to the effects of neuroinflammation in both EAE-induced motor deficits [21] and anxiety (present study). CB1-KO mice lack the inhibitory function of CB1Rs in controlling both the glutamatergic and the GABAergic transmission and the effect of pro-inflammatory cytokines on neurons [21, 33, 60], predisposing them to be more sensitive to EAE induction. In fact, pharmacological activation of CB1R dampens the TNF-mediated potentiation of striatal spontaneous glutamate-mediated excitatory postsynaptic currents (sEPSCs), which is believed to cogently contribute to the inflammation-induced neurodegenerative damage observed in EAE mice. Furthermore, mice lacking CB1R showed a more severe clinical course and, in parallel, exacerbated alterations of sEPSC duration after induction of EAE, indicating that endogenous cannabinoids activate CB1R and mitigate the synaptotoxic action of TNF in EAE [21].

The interaction between IL1β and the ECS is also emerging, based on the evidence that IL-1β effects on striatal spontaneous excitatory and inhibitory currents are regulated by transient receptor potential vanilloid 1 (TRPV1) channels, members of the ECS [61]. Furthermore, IL-1β has also been shown to modulate the sensitivity of CB1Rs controlling synaptic transmission in the striatum ([62], present study]). Of note, IL-1β is involved in mood alterations associated with inflammatory illnesses and with stress. In line with this, a single icv injection of IL-1β caused anxiety in mice and abrogated the sensitivity of CB1Rs controlling GABA synapses in the striatum. Identical behavioral and synaptic results were obtained following social defeat stress, and icv injection of IL-1ra reverted both effects [33]. The present findings reported in EAE are consistent with our previous observations and reinforce them. In fact, both behavioral and synaptic dysfunctions were abrogated by in vivo inhibition of IL1-β.

IL-1β-dependent inhibition of striatal CB1R function was mediated by the interference of this pro-inflammatory cytokine with the DA system, since DAergic stimulation with amphetamine abrogated the effects of EAE on CB1Rs, and IL-1ra reversed the biochemical and molecular [10], as well as the behavioral defects and [10, present study] of DA transmission.

The role of DA in the control of striatal CB1R function has already been reported in previous studies, showing that in vivo facilitation of DA release with cocaine enhances the sensitivity of these receptors [13], while DA receptor blockade with haloperidol causes the opposite effect [49]. Interestingly, the role of dopamine in immune function and fatigue perception in MS is also emerging and other studies are needed to explore the mechanisms of dopamine imbalance in MS [63]. Here, we found that amphetamine treatment was able to restore normal CB1R functioning at GABA terminals in the striatum of EAE mice, confirming the link between the two systems in this brain area. We therefore asked whether amphetamine treatment could also recover mood disturbance in EAE mice, but we could not assess behavior in the treated mice. Indeed, in our experimental condition, about 70 % of the mice showed hypo-locomotion (number of the line crossing between the LD compartments: EAE amphetamine: 1.58 ± 0.47, *n* = 17; EAE vehicle: 3.78 ± 0.42, *n* = 14; unpaired *T* test **p = 0.0021, data not shown), in accordance with the literature reporting that amphetamine affects locomotion in a dose- and time-dependent manner [64, 65].

Since we have previously hypothesized a D1R-D2R altered signaling in the EAE striatum due to increased phosphorylation of DARPP32 at th34 [10], we analyzed the effect of amphetamine on this biochemical feature of the DA system in the EAE striatum. Amphetamine as well as D1R agonists is reported to induce a rapid increase in the phosphorylation of DARPP32 at Th34 site (15 or 30 min after injection) [37, 66], by activating D1R and inducing a potentiation of the PKA pathway. In our experimental settings, we found that in control mice, amphetamine did not change the phosphorylation status of DARPP32, possibly due to the later time-point of evaluation (24 h) and the dose administered. However, amphetamine induced an upregulation of the total DARPP32, which accounted for the reduction of the DARPP32 phosphorylation compared to EAE. In the light of the reduced D2R signaling in the striatum of EAE mice [10], we suppose that the increased DA levels due to amphetamine treatment restore D2Rs functioning counteracting the aberrant condition of D1 oversensitization in EAE. This ultimately induces compensatory mechanisms aimed at enhancing DARPP32 availability for phosphorylation.

Our electrophysiological recordings suggest that increasing DA levels induces presynaptic rearrangements that recover CB1R function on GABA terminals. Thus, we explored possible mechanisms to explain this result. One possibility was given by the interaction between D2R and CB1R mediated by BDNF as described in a previous study: D2R stimulation induces BDNF downregulation, which in turn regulates CB1R function in striatum [49]. The proposed mechanism was linked to the location of both kinds of receptors at lipid raft. In line with the hypothesis of D2R-CB1R desensitization in the EAE striatum, we found for the first time that BDNF mRNA is increased in the striatum of EAE mice. However, amphetamine treatment recovers CB1R function in a BDNF-independent manner. Another possibility was given by the phosphorylation of CB1R at ser316. Although the role of CB1R phosphorylation in the endocannabinoid-mediated neurotransmission is poorly studied, it has been proposed that it regulates the internalization of the receptor-ligand complex [53], a process necessary for the normal functioning of the receptor signaling. Upregulation of CB1R phosphorylation has been interpreted as a self-regulating mechanism to reduce CB1R signaling [67] and linked to increased social play [68].

Here, we found that CB1R phosphorylation is reduced in the striatum of EAE mice, indicating an altered CB1R signaling and functioning and corroborating the electrophysiological results. Amphetamine treatment was not able to restore normal phosphorylation status of CB1R, leading to the conclusion that also this mechanism was not involved in amphetamine-induced recovery of CB1R sensitivity on GABA synapses.

It is worth mentioning that the D2R-CB1R signaling is the subject of extensive investigation. Several other mechanisms, here not explored, may take place, such as the formation of D2R-CB1R heteromers or the involvement of CB1R accessory proteins, like the cannabinoid receptor interacting protein type 1 (CRIP1) [69]. Although the results of the present investigation do not allow exhaustive conclusions about the mechanisms regulating the D2R-CB1R signaling in the EAE striatum, they clearly indicate that in the EAE striatum, the DA system is compromised and that this affect the function of CB1R.

## Conclusions

Collectively, our data contribute to clarify the synaptic and, at least in part, molecular basis of mood disturbances in EAE and, possibly, MS. Understanding the neurobiological underpinning of anxiety-like behavior in EAE mice is of crucial importance to optimize the treatment of mood disturbance in MS and, possibly, other neuroinflammatory diseases. In this direction, targeting the endocannabinoid system may be a valid therapeutic tool for the treatment of both psychiatric and motor symptoms in MS patients.

## Abbreviations

ACSF: Artificial CSF
BDNF: Brain-derived neurotrophic factor
CB1R: Type-1 cannabinoid receptor
CFA: Complete Freund’s adjuvant
CRIP1: Cannabinoid receptor interacting protein type 1
CSF: Cerebrospinal fluid
DA: Dopamine
DARPP32: DA- and cAMP-regulated phosphoprotein 32 kDa
dpi: Days post immunization
EAE: Experimental autoimmune encephalomyelitis
ECS: Endocannabinoid system
icv: Intracerebroventricular
IL1-ra: Interleukin 1 receptor antagonist
IL-1β: Interleukin-1β
LDT: Light/dark test
MOG_35-55_: Myelin oligodendrocyte glycoprotein 35-55
MS: Multiple sclerosis
MSNs: Medium spiny projection neurons
NB: Nest building
PBS: Phosphate-buffered saline
sEPSCs: Spontaneous excitatory postsynaptic currents
sIPSCs: Spontaneous inhibitory postsynaptic currents
TNF: Tumor necrosis factor
TRPV1: Transient receptor potential vanilloid 1 channels
WB: Western blot
WT: Wild type

## References

[1] Haussleiter IS, Brüne M, Juckel G (2009). Psychopathology in multiple sclerosis: diagnosis, prevalence and treatment. Ther Adv Neurol Disord.

[2] Lo Fermo S, Barone R, Patti F, Laisa P, Cavallaro TL, Nicoletti A (2010). Outcome of psychiatric symptoms presenting at onset of multiple sclerosis: a retrospective study. Mult Scler.

[3] Imitola J, Chitnis T, Khoury SJ (2005). Cytokines in multiple sclerosis: from bench to bedside. Pharmacol Ther.

[4] Maimone D, Gregory S, Arnason BG, Reder AT (1991). Cytokine levels in the cerebrospinal fluid and serum of patients with multiple sclerosis. J Neuroimmunol.

[5] Kahl KG, Kruse N, Faller H, Weiss H, Rieckmann P (2002). Expression of tumor necrosis factor-alpha and interferon-gamma mRNA in blood cells correlates with depression scores during an acute attack in patients with multiple sclerosis. Psychoneuroendocrinology.

[6] Rossi S, Motta C, Studer V, Macchiarulo G, Volpe E, Barbieri F (2014). Interleukin-1β causes excitotoxic neurodegeneration and multiple sclerosis disease progression by activating the apoptotic protein p53. Mol Neurodegener.

[7] Pollak Y, Ovadia H, Orion E, Weidenfeld J, Yirmiya R (2003). The EAE-associated behavioral syndrome: I. Temporal correlation with inflammatory mediators. J Neuroimmunol.

[8] Haji N, Mandolesi G, Gentile A, Sacchetti L, Fresegna D, Rossi S (2012). TNF-α-mediated anxiety in a mouse model of multiple sclerosis. Exp Neurol Elsevier Inc.

[9] Acharjee S, Nayani N, Tsutsui M, Hill MN, Ousman SS, Pittman QJ (2013). Altered cognitive-emotional behavior in early experimental autoimmune encephalitis—cytokine and hormonal correlates. Brain Behav Immun.

[10] Gentile A, Fresegna D, Federici M, Musella A, Rizzo FR, Sepman H (2015). Dopaminergic dysfunction is associated with IL-1β-dependent mood alterations in experimental autoimmune encephalomyelitis. Neurobiol Dis.

[11] Katona I, Sperlágh B, Sík A, Käfalvi A, Vizi ES, Mackie K (1999). Presynaptically located CB1 cannabinoid receptors regulate GABA release from axon terminals of specific hippocampal interneurons. J Neurosci.

[12] Domenici MR, Azad SC, Marsicano G, Schierloh A, Wotjak CT, Dodt HU (2006). Cannabinoid receptor type 1 located on presynaptic terminals of principal neurons in the forebrain controls glutamatergic synaptic transmission. J Neurosci.

[13] Centonze D, Rossi S, De Chiara V, Prosperetti C, Battista N, Bernardi G (2007). Chronic cocaine sensitizes striatal GABAergic synapses to the stimulation of cannabinoid CB1 receptors. Eur J Neurosci.

[14] Centonze D, Rossi S, Prosperetti C, Gasperi V, De Chiara V, Bari M (2007). Endocannabinoids limit metabotropic glutamate 5 receptor-mediated synaptic inhibition of striatal principal neurons. Mol Cell Neurosci.

[15] Tao G, Datta S, He R, Nelson F, Wolinsky JS, Narayana PA (2009). Deep gray matter atrophy in multiple sclerosis: a tensor based morphometry. J Neurol Sci.

[16] Baker D, Pryce G, Croxford JL, Brown P, Pertwee RG, Makriyannis A (2001). Endocannabinoids control spasticity in a multiple sclerosis model. FASEB J.

[17] Baker D, Pryce G (2008). The endocannabinoid system and multiple sclerosis. Curr Pharm Des.

[18] Zhang H, Hilton DA, Hanemann CO, Zajicek J (2011). Cannabinoid receptor and N-acyl phosphatidylethanolamine phospholipase D-evidence for altered expression in multiple sclerosis. Brain Pathol.

[19] Lutz B, Marsicano G, Maldonado R, Hillard CJ (2015). The endocannabinoid system in guarding against fear, anxiety and stress. Nat Rev Neurosci.

[20] Pryce G, Ahmed Z, Hankey DJR, Jackson SJ, Croxford JL, Pocock JM (2003). Cannabinoids inhibit neurodegeneration in models of multiple sclerosis. Brain.

[21] Rossi S, Furlan R, De Chiara V, Muzio L, Musella A, Motta C (2011). Cannabinoid CB1 receptors regulate neuronal TNF-α effects in experimental autoimmune encephalomyelitis. Brain Behav Immun.

[22] Ramil E, Sánchez AJ, González-Pérez P, Rodríguez-Antigüedad A, Gómez-Lozano N, Ortiz P (2010). The cannabinoid receptor 1 gene (CNR1) and multiple sclerosis: an association study in two case-control groups from Spain. Mult Scler.

[23] Rossi S, Buttari F, Studer V, Motta C, Gravina P, Castelli M (2011). The (AAT)n repeat of the cannabinoid CB1 receptor gene influences disease progression in relapsing multiple sclerosis. Mult Scler.

[24] Rossi S, Bozzali M, Bari M, Mori F, Studer V, Motta C (2013). Association between a genetic variant of type-1 cannabinoid receptor and inflammatory neurodegeneration in multiple sclerosis. PLoS One.

[25] Hariri AR, Gorka A, Hyde LW, Kimak M, Halder I, Ducci F (2009). Divergent effects of genetic variation in endocannabinoid signaling on human threat- and reward-related brain function. Biol Psychiatry.

[26] Patel S, Hillard CJ (2006). Pharmacological evaluation of cannabinoid receptor ligands in a mouse model of anxiety: further evidence for an anxiolytic role for endogenous cannabinoid. J Pharmacol Exp.

[27] Micale V, Cristino L, Tamburella A, Petrosino S, Leggio GM, Drago F (2009). Anxiolytic effects in mice of a dual blocker of fatty acid amide hydrolase and transient receptor potential vanilloid type-1 channels. Neuropsychopharmacology.

[28] Hill MN (2009). Impairments in endocannabinoid signaling and depressive illness. JAMA.

[29] Topol EJ, Bousser MG, Fox KA, Creager MA, Despres JP, Easton JD (2010). Rimonabant for prevention of cardiovascular events (CRESCENDO): a randomised, multicentre, placebo-controlled trial. Lancet.

[30] Jacob W, Yassouridis A, Marsicano G, Monory K, Lutz B, Wotjak CT (2009). Endocannabinoids render exploratory behaviour largely independent of the test aversiveness: role of glutamatergic transmission. Genes Brain Behav.

[31] Beyer CE, Dwyer JM, Piesla MJ, Platt BJ, Shen R, Rahman Z (2010). Depression-like phenotype following chronic CB1 receptor antagonism. Neurobiol Dis.

[32] De Chiara V, Errico F, Musella A, Rossi S, Mataluni G, Sacchetti L (2010). Voluntary exercise and sucrose consumption enhance cannabinoid CB1 receptor sensitivity in the striatum. Neuropsychopharmacology.

[33] Rossi S, Sacchetti L, Napolitano F, De Chiara V, Motta C, Studer V (2012). Interleukin-1 causes anxiety by interacting with the endocannabinoid system. J Neurosci.

[34] Centonze D, Muzio L, Rossi S, Cavasinni F, De Chiara V, Bergami A (2009). Inflammation triggers synaptic alteration and degeneration in experimental autoimmune encephalomyelitis. J Neurosci.

[35] Marsicano G, Wotjak CT, Azad SC, Bisogno T, Rammes G, Cascio MG (2002). The endogenous cannabinoid system controls extinction of aversive memories. Nature.

[36] Mandolesi G, Musella A, Gentile A, Grasselli G, Haji N, Sepman H (2013). Interleukin-1β alters glutamate transmission at purkinje cell synapses in a mouse model of multiple sclerosis. J Neurosci.

[37] Napolitano F, Bonito-Oliva A, Federici M, Carta M, Errico F, Magara S (2010). Role of aberrant striatal dopamine D1 receptor/cAMP/protein kinase A/DARPP32 signaling in the paradoxical calming effect of amphetamine. J Neurosci.

[38] Crawley JN (1981). Neuropharmacologic specificity of a simple animal model for the behavioral actions of benzodiazepines. Pharmacol Biochem Behav.

[39] Paumier KL, Sukoff Rizzo SJ, Berger Z, Chen Y, Gonzales C, Kaftan E (2013). Behavioral characterization of A53T mice reveals early and late stage deficits related to Parkinson’s disease. PLoS One.

[40] Bulloch K, Hamburger RN, Loy R (1982). Nest-building behavior in two cerebellar mutant mice: staggerer and weaver. Behav Neural Biol.

[41] Musella A, De Chiara V, Rossi S, Prosperetti C, Bernardi G, Maccarrone M (2009). TRPV1 channels facilitate glutamate transmission in the striatum. Mol Cell Neurosci.

[42] Aubert A (1999). Sickness and behaviour in animals: a motivational perspective. Neurosci Biobehav Rev.

[43] Blossom SJ, Doss JC, Hennings LJ, Jernigan S, Melnyk S, James SJ (2008). Developmental exposure to trichloroethylene promotes CD4+ T cell differentiation and hyperactivity in association with oxidative stress and neurobehavioral deficits in MRL+/+ mice. Toxicol Appl Pharmacol.

[44] Pollak Y, Ovadia H, Goshen I, Gurevich R, Monsa K, Avitsur R (2000). Behavioral aspects of experimental autoimmune encephalomyelitis. J Neuroimmunol.

[45] Rossi S, De Chiara V, Musella A, Kusayanagi H, Mataluni G, Bernardi G (2008). Chronic psychoemotional stress impairs cannabinoid-receptor-mediated control of GABA transmission in the striatum. J Neurosci.

[46] Centonze D, Bari M, Rossi S, Prosperetti C, Furlan R, Fezza F (2007). The endocannabinoid system is dysregulated in multiple sclerosis and in experimental autoimmune encephalomyelitis. Brain.

[47] Mori F, Nisticò R, Mandolesi G, Piccinin S, Mango D, Kusayanagi H (2013). Interleukin-1β promotes long-term potentiation in patients with multiple sclerosis. NeuroMolecular Med.

[48] Rossi S, De Chiara V, Musella A, Mataluni G, Sacchetti L, Bernardi G (2009). Adaptations of striatal endocannabinoid system during stress. Mol Neurobiol.

[49] De Chiara V, Angelucci F, Rossi S, Musella A, Cavasinni F, Cantarella C (2010). Brain-derived neurotrophic factor controls cannabinoid CB1 receptor function in the striatum. J Neurosci.

[50] Picada JN, Dos Santos Bde J, Celso F, Monteiro JD, Da Rosa KM, Camacho LR (2011). Neurobehavioral and genotoxic parameters of antipsychotic agent aripiprazole in mice. Acta Pharmacol Sin.

[51] Babovic D, Jiang L, Goto S, Gantois I, Schütz G, Lawrence AJ (2013). Behavioural and anatomical characterization of mutant mice with targeted deletion of D1 dopamine receptor-expressing cells: response to acute morphine. J Pharmacol Sci.

[52] Luque-Rojas MJ, Galeano P, Suárez J, Araos P, Santín LJ, de Fonseca FR (2013). Hyperactivity induced by the dopamine D2/D3 receptor agonist quinpirole is attenuated by inhibitors of endocannabinoid degradation in mice. Int J Neuropsychopharmacol.

[53] Daigle TL, Kwok ML, Mackie K (2008). Regulation of CB1 cannabinoid receptor internalization by a promiscuous phosphorylation-dependent mechanism. J Neurochem.

[54] Mathew SJ, Ho S (2006). Etiology and neurobiology of social anxiety disorder. J Clin Psychiatry.

[55] Révy D, Jaouen F, Salin P, Melon C, Chabbert D, Tafi E (2014). Cellular and behavioral outcomes of dorsal striatonigral neuron ablation: new insights into striatal functions. Neuropsychopharmacology.

[56] Marrie R, Horwitz R, Cutter G, Tyry T, Campagnolo D, Vollmer T (2009). The burden of mental comorbidity in multiple sclerosis: frequent, underdiagnosed, and undertreated. Mult Scler.

[57] Maes M, Meltzer HY, Bosmans E, Bergmans R, Vandoolaeghe E, Ranjan R (1995). Increased plasma concentrations of interleukin-6, soluble interleukin-6, soluble interleukin-2 and transferrin receptor in major depression. J Affect Disord.

[58] Zorrilla EP, Luborsky L, McKay JR, Rosenthal R, Houldin A, Tax A (2001). The relationship of depression and stressors to immunological assays: a meta-analytic review. Brain Behav Immun.

[59] Dantzer R, O’Connor JC, Freund GG, Johnson RW, Kelley KW (2008). From inflammation to sickness and depression: when the immune system subjugates the brain. Nat Rev Neurosci.

[60] Rossi S, Motta C, Musella A, Centonze D (2015). The interplay between inflammatory cytokines and the endocannabinoid system in the regulation of synaptic transmission. Neuropharmacology.

[61] Musumeci G, Grasselli G, Rossi S, De Chiara V, Musella A, Motta C (2011). Transient receptor potential vanilloid 1 channels modulate the synaptic effects of TNF-α and of IL-1β in experimental autoimmune encephalomyelitis. Neurobiol Dis.

[62] De Chiara V, Motta C, Rossi S, Studer V, Barbieri F, Lauro D (2013). Interleukin-1β alters the sensitivity of cannabinoid CB1 receptors controlling glutamate transmission in the striatum. Neuroscience.

[63] Dobryakova E, Genova HM, DeLuca J, Wylie GR (2015). The dopamine imbalance hypothesis of fatigue in multiple sclerosis and other neurological disorders. Front Neurol.

[64] Yates JW, Meij JTA, Sullivan JR, Richtand NM, Yu L (2007). Bimodal effect of amphetamine on motor behaviors in C57BL/6 mice. Neurosci Lett.

[65] Spielewoy C, Biala G, Roubert C, Hamon M, Betancur C, Giros B (2002). Hypolocomotor effects of acute and daily d-amphetamine in mice lacking the dopamine transporter. Psychopharmacology (Berl).

[66] Bibb JA, Snyder GL, Nishi A, Yan Z, Meijer L, Fienberg AA (1999). Phosphorylation of DARPP-32 by Cdk5 modulates dopamine signalling in neurons. Nature.

[67] Garcia DE, Brown S, Hille B, Mackie K (1998). Protein kinase C disrupts cannabinoid actions by phosphorylation of the CB1 cannabinoid receptor. J Neurosci.

[68] Trezza V, Damsteegt R, Manduca A, Petrosino S, Van Kerkhof LW, Pasterkamp RJ (2012). Endocannabinoids in amygdala and nucleus accumbens mediate social play reward in adolescent rats. J Neurosci.

[69] Smith TH, Blume LC, Straiker A, Cox JO, David BG, McVoy JRS (2015). Cannabinoid receptor-interacting protein 1a modulates CB1 receptor signaling and regulation. Mol Pharmacol.

